# Influence of Deposition Temperature on Microstructure and Properties of Tantalum Oxide Sputtered Coatings

**DOI:** 10.3390/ma18091895

**Published:** 2025-04-22

**Authors:** Maria P. Nikolova, Iliyan Tzvetkov

**Affiliations:** Department of Material Science and Technology, University of Ruse “Angel Kanchev”, 8 Studentska Str., 7017 Ruse, Bulgaria; itzvetkov@uni-ruse.bg

**Keywords:** magnetron sputtering, tantalum oxide, bio-coatings, thin films, Ti6Al4V implants, corrosion, wear

## Abstract

To increase the wear and corrosion resistance of (α + β) titanium-aluminium-vanadium (Ti6Al4V) alloy, ceramic tantalum oxide coatings were deposited by direct current (DC) magnetron sputtering at three different substrate temperatures—400, 450, and 500 °C. The crystallographic structure, surface morphology, chemical compositions, film adhesion, and hardness of the coatings were described using XRD, SEM, EDS, scratch tests, and microhardness measurements. The samples’ ability to withstand corrosion was assessed using electrochemical studies. Results revealed that thin films have an amorphous or crystalline structure dependent on temperature. The film’s thicknesses varied between 560 and 600 nm. With the increase in deposition temperature, the hardness of the film rose. All oxide coatings were tightly adherent to the titanium alloy substrate, and critical force increased from about 8.6 up to 20 N when the temperature rose from 400 to 500 °C. During the polarisation investigations, after 1 h of immersion, a drop in current density (j_corr_) verified an improvement in the corrosion resistance of the amorphous and well-crystalline coatings. A two-layer model of the surface film accurately describes the coated systems’ electrochemical behaviour. However, according to the EIS analysis, the well-crystalline film deteriorates greatly, whereas the amorphous film prevents penetration during the 7-day immersion test in SBF. The wettability tests demonstrated the hydrophilic nature of the coatings, and after seven days, the mineralisation of calcium phosphate proves the coatings become bioactive in simulated bodily fluid (SBF). Thus, we produced films of tantalum oxide, which, with the proper deposition parameters, may prove to be appropriate surfaces for titanium-based implant bio-applications.

## 1. Introduction

The biomaterials industry has grown quickly to meet the needs of an ageing population since biomaterials contribute to longer lifespans and better quality of life for people. The metals utilised as biomaterials are long-term fixtures with exceptional mechanical strength, appropriate for load-bearing applications, and can help replace or repair bone tissue. Because of its advantageous properties in terms of specific strength, chemical stability, corrosion resistance, cytocompatibility, and improved osseointegration, Ti6Al4V alloy is one of the preferred materials for permanent biomaterials, especially for dental and orthopaedic applications [1,2]. When titanium metal is exposed to air at ambient temperature, a thin oxide surface layer (1.5–10 nm) naturally forms, which increases the surface stability [3]. Nevertheless, Ti possesses biological inertia [4], which causes weak and sluggish osseointegration, particularly when diabetes or osteoporosis is present [5]. Additionally, in load-bearing applications, Ti alloys have poor wear resistance and produce wear debris that can cause bone resorption, loosening, and failure, in addition to contaminating the surrounding tissue [6]. Furthermore, cytotoxicity may arise from the emission of aluminium and vanadium at high friction levels [7]. Vanadium causes damaging tissue reactions, and the release of both V and Al ions is linked to chronic illnesses like Parkinson’s and Alzheimer’s diseases as well as peripheral neuropathy [8]. Therefore, applying coatings on implant surfaces is seen to be a practical tactic to address these issues since the materials’ corrosion and wear characteristics are strongly correlated with their surface chemical and physical characteristics, microstructure, and composition.

Because of their exceptional resistance to wear and corrosion, transition metal oxide films have been used during the past few decades to enhance mechanical and corrosion characteristics of biomedical components [9]. Tantalum (Ta) is highly desirable for biomedical applications because of its strong chemical stability and outstanding biocompatibility [10]. Compared to commercially pure Ti (cpTi) and Ti6Al4V alloys, tantalum is a non-toxic element [11] and is more compatible with bone tissue [12]. Ta has been used in surgical applications for fixation plates, cranial plates, wire mesh, and suture wire [13]. However, its applications are limited by high cost and difficulties in creating dense Ta implants [14]. Ta easily creates a stable protective layer and establishes strong connections with oxygen atoms. Because of their strong covalent bonding, tantalum oxide coatings offer great hardness and chemical durability, making them good options for bioactive coatings. Tantalum oxide coatings have thus been applied to several materials to enhance their surface characteristics. It was established that ceramic tantalum oxide thin film has improved the mechanical properties and corrosion resistance of Ti6Al4V substrate [15,16]. According to Almeida Alves et al., DC sputtered Ta_1−x_O_x_ films exhibit greater rates of hydroxyapatite (HAp) production in SBF than Ta, which may result in improved osseointegration and bioactivity [17]. Layers of TaO have been demonstrated to increase cytocompatibility; for instance, in vitro experiments have shown that the coatings encourage proliferation, alkaline osteogenic gene expressions of osteoblasts, mineralisation, and phosphatase (ALP) activity [18]. Concerning chemical stability, according to earlier research [19], the presence of high-valence tantalum oxides, such as Ta_2_O_5_, stabilises a passive surface which lowers the rate of corrosion. Moreover, according to Beline T. et al. [20], for biological applications, a crystalline β-Ta_2_O_5_ film proved to favourably impact cell morphology and spreading compared to cpTi. Moreover, excellent antimicrobial activity against Gram-negative oral pathogens like *P. gingivalis* and *F. nucleatum* was demonstrated using Ta-based coatings that contained Ta_2_O_5_ [21]. Similar findings were reported by Chang et al. [22], who displayed that amorphous tantalum oxide coatings decreased the adhesion of *Streptococcus aureus* and *Actinobacillus actinomycetemcomitans*, while the crystalline tantalum oxide (β-Ta_2_O_5_) coatings demonstrated superior cellular biocompatibility with human skin fibroblasts. Therefore, Ta_2_O_5_ is an appropriate substitute for use as a protective coating due to its biocompatibility, corrosion resistance, bactericidal properties, and abrasion resistance.

Different surface treatments have recently been the subject of extensive research to enhance the mechanical characteristics and corrosion behaviour of various alloys. For tantalum-containing coating, PVD (physical vapour deposition), CVD (chemical vapour deposition), electrochemical deposition, sol-gel, metal-organic decomposition, plasma-enhanced atomic layer deposition (PEALD), pulsed laser deposition (PLD), and laser-engineering net shaping (LENS) are the most widely used techniques [23,24,25,26,27,28]. Because it can improve the alloy surface qualities, magnetron sputtering (MS) technology is one of the most popular PVD processes for biomedical coatings [29,30]. Sputtering saves money and produces extremely uniform and pure thin coatings that stick firmly to a substrate over wide areas [31]. The sputtering power and duration, deposition distance, temperature, gas pressure and composition, substrate surface quality, and other process variables all have a direct impact on the coatings’ phase composition, structure, and properties. Thus, tuning the process parameters with good reproducibility allows for obtaining a variety of film characteristics. Then, new opportunities for developing alternative bone grafts are opened by coupling the capabilities of the bioactive T_2_O_5_ coating with the superior tribological and corrosion properties and the mechanical strength of titanium alloys as a foundation material for implants.

Due to the extremely high melting temperature of bulk tantalum (3017 °C), the tantalum oxide coating’s crystalline state is directly correlated with the preparation conditions’ temperature [32]. Since TaO_x_ thin film crystallisation is difficult [33], most magnetron studies produce amorphous oxides that are then crystallised by high-temperature (700–900 °C) post-deposition annealing [22,34]. However, because the substrate and coating have different thermal expansion coefficients, post-heat treatment may weaken the bond between them [35]. Additionally, the interaction of Ta with oxygen happens very quickly and results in a rise in volume [36]. However, to our knowledge, limited research has been carried out on the direct acquisition of tantalum oxide coatings during magnetron sputtering at various substrate temperatures. For example, Sahoo and his colleagues [37] produced radio frequency (RF) sputtered Ta_2_O_5_ films by varying the substrate temperature from 100 to 300 °C to optimise their electrical properties. However, the impact of a higher substrate temperature during reactive DC magnetron sputtering has not been investigated. Consequently, in this work, we used DC reactive magnetron sputtering to deposit tantalum oxide films onto Ti6Al4V substrates at temperatures varying between 400 and 500 °C. X-ray diffraction (XRD) and scanning electron microscopy (SEM) analyses were utilised to evaluate the coatings’ chemistry and phase composition. The changes in electrochemical impedance (EIS), pH, solution ion content, and films’ mineralisation were determined by real-time in vitro immersion tests in simulated body fluid (SBF) solution.

## 2. Materials and Methods

### 2.1. Substrate Material and Initial Preparation

Samples of 14 × 14 × 2 mm Ti6Al4V alloy were subject to tantalum oxide deposition. Silicon carbide (SiC) sandpaper sheets with grit sizes ranging from 220 to 4000 were used to grind the surface of the plate samples. After polishing, they were then sonicated in ethanol for five minutes and warm air-dried.

### 2.2. Coating Deposition

The evacuation of the cubic vacuum chamber with water-cooled walls resulted in a base pressure of 3 × 10^−2^ Pa. To increase the adherence of the oxide coating before the DC magnetron deposition, the samples were glow discharge cleaned in a pure Ar atmosphere at a pressure of 8 Pa, target current of 1 A for 15 min at the process substrate temperature. The deposition was performed in a pure oxygen atmosphere at a pressure of 1.8 × 10^−1^ Pa and using a Ta target of 99.95% purity. The target current was kept equal to 1.3 A (300 V) for a deposition time of 240 min. A negative bias of 100 V was applied to the substrates while the substrate temperature was varied at 400, 450, and 500 °C. The distance between the target and the sample was 50 mm, and the sample stage was made to rotate at a speed of 0.33 rpm to ensure that the coatings’ composition remained constant. Additionally, to measure the thickness of the layer, deposits on glass slides were also tested. Based on earlier pilot tests, the mentioned technological settings proved the most appropriate after extensive testing of the coatings’ morphology, stability, thickness, and adhesion at acceptable deposition rates and uniformity.

### 2.3. Characterisation

A scanning electron microscope (SEM, LYRA I XMU, Tescan, Brno, Czech Republic) was used to assess the deposits’ surface composition by energy dispersive spectrometer (EDS, Quantax 200, Bruker, Billerica, MA, USA) and morphology on the substrate and coatings before and after immersion tests. To determine the thickness of each layer, SEM images of the cross-section of a mechanically fractured glass specimen coated with titanium samples were also obtained.

Symmetrical Bragg–Brentano mode and Ni-filtered CuK_α_ radiation (λ = 0.154178 nm) were employed to analyse the phase composition using X-ray diffraction (XRD, URD-6 Seifert & Co., Antweiler, Germany) in a 2θ range of 20–80° and a step of 0.1°. The scanning rate was 6 s per step.

Coating adhesion was tested using a CSEM Revetest^®^ scratch tester (CSM Instruments SA, Peseux, Switzerland) with an optical microscope and a conventional Rockwell-C diamond indenter (cone with a 120° apex angle and a 200 μm tip radius) up to a normal load of 30 N. Scratch track images and an abrupt change in the coefficient of friction, which indicate coating fracture information, were used to calculate the critical load F_c_. To determine the coefficient of friction (COF), a sliding wear study was conducted using the CSEM Revetest^®^ scratch tester’s diamond indenter (normal load 8 N, linear speed 0.3 mm/s, testing period 30 s). Room temperature and open-air conditions were used for the trials. An average of the replicated friction test findings is presented after each test was conducted three times.

Using a Knoop indenter and load control mode, a DuraScan 20 Lite (ZwickRoell GmbH Co. KG, Ulm, Germany) tester was utilised to quantify the microhardness at a peak force of 5 gf. To collect statistical data and determine the dispersion of the results, ten measurements were taken on each polished and coated surface.

The simulated bodily fluid (SBF) solution was made by dissolving 8.035 g NaCl, 0.355 g NaHCO_3_, 0.225 g KCl, 0.176 g K_2_HPO_4_·3H_2_O, 0.311 g MgCl_2_·6H_2_O, 0.388 g CaCl_2_, 0.072 g Na_2_SO_4_, and 6.118 g (CH_2_OH)_3_CNH_2_ (Tris) in 1 L distilled water one after the other. Then, using 1 N HCl at 37 °C, the solution was stabilised at pH 7.4.

A face contact angle setup with a camera and a microscope was used to measure contact angles, applying the sessile drop method. The surface residue was removed using ethanol. A microliter syringe was utilised to suspend a 5.0 μL droplet of SBF from its tip. Until the droplet touched the sample surface, the sample surface was moved closer to the syringe tip. To obtain accurate average readings, 30 s after the probe liquid droplet reached equilibrium, five separate measurements were taken on each surface at room temperature, with a relative humidity of 21%, and recorded on camera. The contact angle values were calculated from the acquired photos using Autodesk AutoCAD 2018. Mean contact angles were measured and documented, along with standard deviation values.

An Interface 1010E potentiostat (Gamry Instruments, Inc., Philadelphia, PA, USA) was used for the experiments in SBF solution. The samples were immersed in 10 millilitres of naturally aerated SBF at 37 ± 0.5 degrees Celsius. Three electrodes were employed in the cell configuration: a saturated calomel reference electrode (SCE), a platinum wire as a counter electrode, and the specimens as working electrodes (0.8 cm^2^ exposed). The open circuit potential (OCP) stabilisation time for the electrochemical studies was one hour. The specimens were polarised to ±0.5 V_SCE_ versus OCP for potentiodynamic polarisation scans, and a scan rate of 1 mV/s was used. After the potentiodynamic polarisation test, the samples were removed, and fresh samples were used to initiate the subsequent electrochemical experiments.

Electrochemical impedance spectroscopy (EIS) was used to examine the surface of bare substrates and coated samples following 1 h and 7 days of immersion in SBF. The EIS spectra were obtained over a frequency range of 10^4^ Hz to 10^−2^ Hz and a ±5 mV RMS sinusoidal perturbation (vs. OCP) was applied. Gamry Echem Analyst software version 7.10 (Gamry Instruments, Inc., Warminster, PA, USA) was used to analyse the electrochemical data collected from EIS. The majority of EIS measurements were made in triplicate.

In parallel tests, the bioactivity assessment was performed in a 10 mL SBF solution. The concentration of Mg and Ca ions before and after 7 days of immersion was tested spectrophotometrically (HI83300, Hanna Instruments, Sat Nusfalau, Romania) from each sample solution. pH values (HI8424, Hanna Instruments, Sat Nusfalau, Romania) were determined periodically for each sample solution. Samples were rinsed with distilled water and allowed to dry at room temperature after each soaking period was completed. SEM/EDS analysis was used to assess the microstructural changes and bone-like apatite layer’s precipitation on each coated and bare surface.

## 3. Results and Discussions

### 3.1. Structural and Chemical Characterisation of the Deposited Films

**Figure 1:**
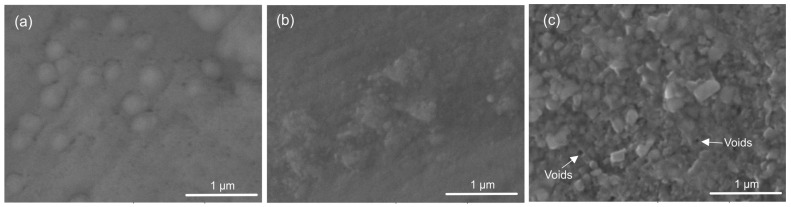
Representative SEM micrographs of the surface morphology of the tantalum oxide coating: (**a**) 400 °C, (**b**) 450 °C, and (**c**) 500 °C of deposition.

The top view of the thin tantalum oxide films is shown in Figure 1. All coatings demonstrated integrity and good adherence to the substrates. Deposited at 400 °C (Figure 1a), the coating’s topography exhibits small spherical-like peaks, showing neither grain boundaries nor crystallites. These spherical clusters seem well adherent to one another. The thin films grown at 450 °C (Figure 1b) represent small crystallised grains with some clustered areas of agglomerated formations. The grains’ size varies and the granular structure is not fully mature. The growing number of tiny, distinct crystallites indicates that the crystallisation is progressing. At a higher temperature (500 °C, Figure 1c), the morphology of the coating suggests the presence of larger crystallites with polycrystalline growth and few pores. At a substrate temperature of 400, 450, and 500 °C and a tantalum melting point of 3017 °C, the homologous temperature at deposition (T_m_) was about 0.13, 0.15, and 0.17. It follows that all films are in Zone T of the structure zone model (SZM) presented by Thornton but have a poor crystalline structure akin to Zone 1. Raising the substrate’s temperature alters the surface mechanism of the film nucleation and speeds up the energy of the bombardment ions, causing the formation of crystalline structures with more densely packed grains. Atomic motions increased as the temperature rose, and they collided with one another. Nonetheless, it turns out that the films’ formations have surface-located voids and pinholes (Figure 1c).

**Figure 2:**
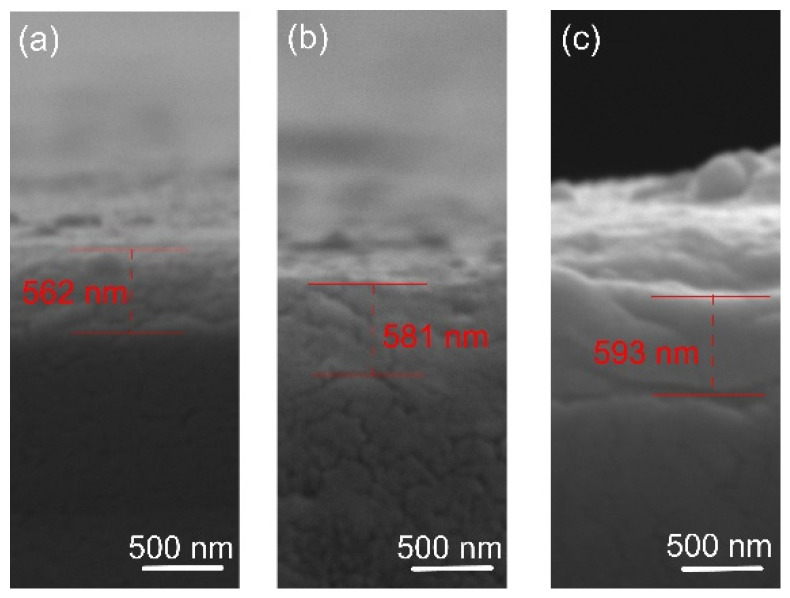
Representative cross-sectional micrographs of the oxide coatings: (**a**) 400 °C, (**b**) 450 °C, and (**c**) 500 °C.

The film thickness was determined by SEM analysis of cross-sections. The results are shown in Figure 2. The coating thickness was found to be about 560, 580, and 590 nm for the films deposited at 400, 450, and 500 degrees, respectively. It is known that the substrate’s temperature barely affects the film’s growth rate [38], as confirmed in this study. The morphology of the film indicates a smooth fractured surface, which is characteristic of the growth of films with small adatom mobility. Similar featureless morphology was reported by [39] when the oxygen flow was increased and film densification occurred. The small thicknesses of the oxide films suggest low deposition rates of Ta. In actuality, when compounds form on the targets, metal sputter rates drastically decrease [40]. The pressure of the reactive gas has a significant impact on this effect. Since a stable stoichiometric ratio of the thin layers must be maintained, increased argon concentration is not preferred over deposition rate, since it was reported that varying oxygen flow levels can trigger a phase shift [39].

The films’ EDS results are shown in Table 1. The Ta-O coatings showed increasing Ta content of about 14.5 at% up to 17.3 at% and decreasing oxygen composition from 61 at% down to 47 at% when increasing the temperature from 400 to 500 °C. A mix of structural, chemical, and process-related factors can account for the O/Ta ratio’s change as the temperature rises. At 400 °C substrate temperature, when the sputtered film is amorphous, the oxygen atoms can be incorporated in excess (interstitially or trapped in voids) since the amorphous structure can tolerate non-stoichiometry. Crystallisation promotes ordering and the rejection of excess oxygen. Moreover, higher temperature also enhances oxygen desorption from weakly bound or interstitial sites and non-stoichiometric, over-oxidised regions. At the temperature increase, the oxygen reacts more efficiently, forming stable oxide bonds (Ta–O), while the volatile oxygen-containing species (such as Ta–O–OH) may desorb or be excluded during crystallisation, which leads to the formation of a more stoichiometric film. Mobile adatoms may disperse or migrate into the spaces between grains, forming either amorphous structures or microscopic pores due to oxygen recombination within the crystal phases.

In addition to Ta and O, Ti is also present in the spectra. The thin film thickness could account for the existence of Ti. The O/Ta ratio of about 2.7 for the highly crystalline coating somewhat exceeds the stoichiometry of Ta_2_O_5_. This phenomenon can be explained by the target surface oxidising when more oxygen is present, which suggests that less Ta can be extracted from the target and fewer Ta–O bonds are formed.

**Figure 3:**
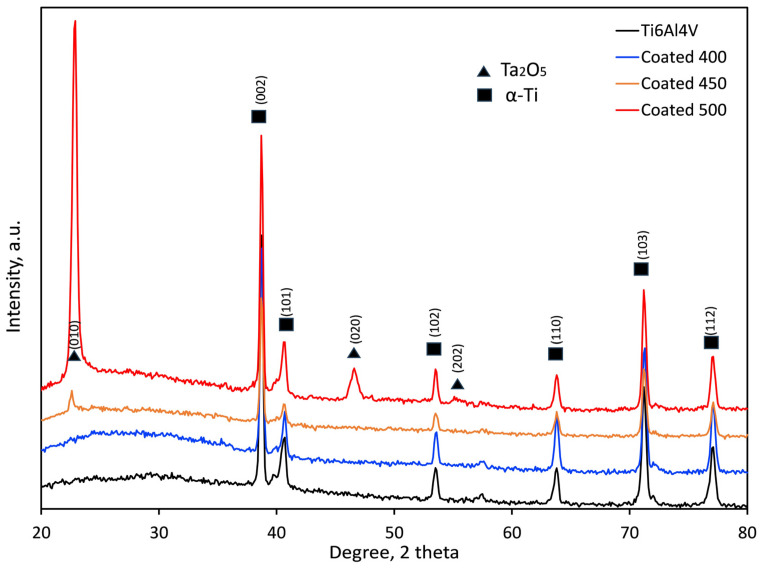
XRD patterns of the bare and coated samples at different temperatures.

The crystallographic structure of the samples was examined by XRD analysis, and the obtained XRD patterns of the coated and control (Ti6Al4V alloy) groups are displayed in Figure 3. Both groups’ diffraction peaks were comparable to metallic α-Ti (International Centre for Diffraction Data (ICDD) card no. 00-001-1198). Cu-K_α_ radiation’s penetration depth (~10 μm), which is greater than the film thickness (≈550–600 nm), explains this behaviour. In addition to α-Ti peaks from the substrate, the XRD pattern of the coated sample indicates the presence of orthorhombic β-Ta_2_O_5_ according to the relevant Joint Committee on Powder Diffraction Standards (JCPDS) card (ICDD card no. 01-079-1375). The broad diffraction maxima (hump) in the 2θ range 20–35° suggests an amorphous structure (a-Ta_2_O_5_) of the coating deposited at 400 °C [23,41,42]. An inadequate energy flux that destabilised the interfacial interaction between tantalum oxide and the substrate material may cause this amorphous layer formation. An amorphous, non-crystalline film was produced when tiny charged clusters nucleated in the gas phase and failed to adjust to the substrate’s surface structure [43].

In contrast, the presence of the well-crystallised orthorhombic structure of β-Ta_2_O_5_ film is shown by the diffraction peaks at 22.8, 46.7, and 55.4° seen in the coated samples at 500 °C. Similar findings on crystallite β-Ta_2_O_5_ peaks have also been documented in Ref. [37]. There is less agglomeration in the gas phase and a chance for a good crystalline structure due to the high-temperature surface-charged clusters [43]. The XRD pattern of the film deposited at 450 °C indicates only a small peak at about 22.8° 2θ, suggesting a fine-grained microstructure of β-Ta_2_O_5_ nanocrystals as confirmed by the SEM image (Figure 1b). By using the Debye–Scherrer equation, the average diffraction domain size was calculated from the width of the diffraction peaks. It was estimated to vary from 21.7 to 28.7 nm for the films on the 450 and 500 °C heated substrates, respectively. The higher the substrate temperature, the stronger the driving force was for the nucleation of β-Ta_2_O_5_. Consequently, it was demonstrated that amorphous, nanocrystalline, and well-crystalline β-Ta_2_O_5_ coatings were successfully created on the titanium alloy substrates.

The substantial increase in the relative intensities of the β-Ta_2_O_5_ diffraction peaks suggests that the crystallinity of the film rises steadily with the increase in deposition temperature from 450 to 500 °C. The (001) peak intensity significantly increases, likely because of the grain growth around the optimal crystallographic orientation.

### 3.2. Mechanical Properties of the Coated Systems

The microhardness values of the coated samples at different temperatures and the bare alloy are displayed in Table 2. It should be noted that the coating thickness, grain size, and residual stresses all impact the hardness of the deposited films [44]. In contrast to the thinner amorphous film, the microhardness of the coating deposited at 450 °C increased as a result of the enlarged interface of the nanograin boundaries, which prevented deformation. The densification and crystallisation of the film with the rise of temperature up to 500 °C additionally increases the hardness values. The surface hardness of Ti6Al4V alloy was boosted by about 85, 113, and 196% when depositing the oxide film at 400, 450, and 500 °C, respectively. Similarly, glow discharge deposited thicker (25 µm) crystalline β-Ta_2_O_5_ coatings on Ti6Al4V alloy demonstrated a 237% increase in surface hardness [45]. In this study, we found that with the rise of deposition temperature and crystallinity of the Ta oxide films, the films’ hardness improves. However, the maximum indentation depth is more than 10% of the coating thickness, which indicates that the substrate’s hardness contributes to the mechanical properties measured.

**Figure 4:**
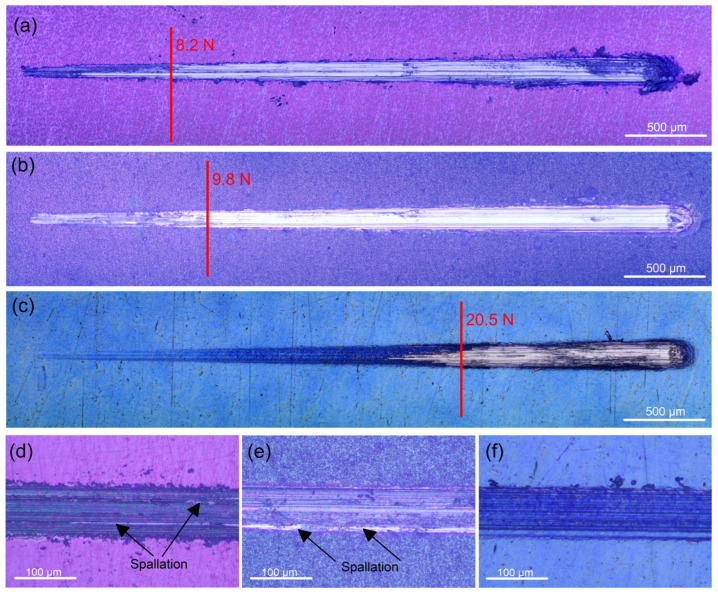
Scratch track images of the tantalum oxide films deposited at: (**a**) 400, (**b**) 450, and (**c**) 500 °C on Ti6Al4V alloy. Representative micrographs of failure after applying a constant load of 8 N on the tantalum oxide films deposited at: (**d**) 400, (**e**) 450, and (**f**) 500 °C.

Since Ti and its alloys have poor wear resistance, protecting Ti implant materials from abrasion brought on by mechanical wear requires proper adhesion between the coating and the substrate. Low residual stress in the interfacial region, high fracture toughness of the materials in contact, and strong atomic contact between the different materials are all indicators of good adhesion. A sharp shift in the coefficient of friction and scratch track images identified the critical load (F_c_) resulting from coating breakdown. After some buckling, at an average critical load of about 8.6 N the 400 °C-deposited coating completely detached from the substrate (Figure 4a). This value is substantially higher than the F_c_ value (1.91 N) reported by Li et al. [46] for ambient temperature sputtered thicker (2.6 µm) Ta_2_O_5_ film with an amorphous structure on the same substrate. The F_c_ value of the 450 °C-deposited film is registered at an average of 9.9 N (Figure 4b), suggesting that the film can withstand a higher load because of the stronger bonding with the substrate. The critical load for the 500 °C-deposited film indicated the highest values (about 20 N), after which the oxide film accumulated at the scratch track’s end, exposing the substrate till the end of the test (Figure 4c). None of the coatings displayed lateral spallation, chipping, or side detachments along the track. This fact confirms that all coatings are not brittle. Because there is less chance of collisions in the gas phase, energetic particle bombardment in lower-temperature sputter deposition produces extremely compressive stresses that can result in adhesive failure at lower normal loads or other unfavourable outcomes [47]. However, the increased deposition temperature reduces the coating growth’s internal stresses, which improves the adhesion strength and film cohesion. Heating the substrate decreases the rate at which impinging particles solidify, improving particle–substrate interaction and, ultimately, film adhesion.

Compared to the uncoated sample, the Ta oxide-coated samples have a coefficient of friction (COF) that is about 12.8, 20.5, and 43.6% lower for the 400, 450, and 500 °C-deposited films, respectively (Table 2). The higher hardness is most likely the cause of this decrease in the coefficient of friction. The Ti6Al4V alloy substrate’s low hardness allowed the indenter to press into it rather easily, causing plastic deformation on the surface of the substrate. On the other hand, greater oxide layer hardness reduced the friction coefficient during the sliding process by preventing plastic deformation. Figure 4d–f display the wear track’s optical micrographs. In contrast to the wear scars of the coatings deposited at 400 and 450 °C temperatures, where the contact with the indenter removes parts of the films and causes spallation within the tracks (Figure 4d,e), the 500 °C-deposited film (Figure 4f) stays intact due to the enhanced wear resistance. Therefore, the well-crystalline surface is a better shield from wear than the amorphous phases of the Ta-oxide coatings.

### 3.3. Wettability

One key factor in determining whether a specimen is suitable for use as a biomedical implant is its surface wettability. The lower contact angle that hydrophilicity implies affects wettability and increases the area of contact with high surface coverage. Wettability has a major impact on the biological reactions of implanted devices because it affects protein absorption and cell activity at the implant surface [48].

**Figure 5:**
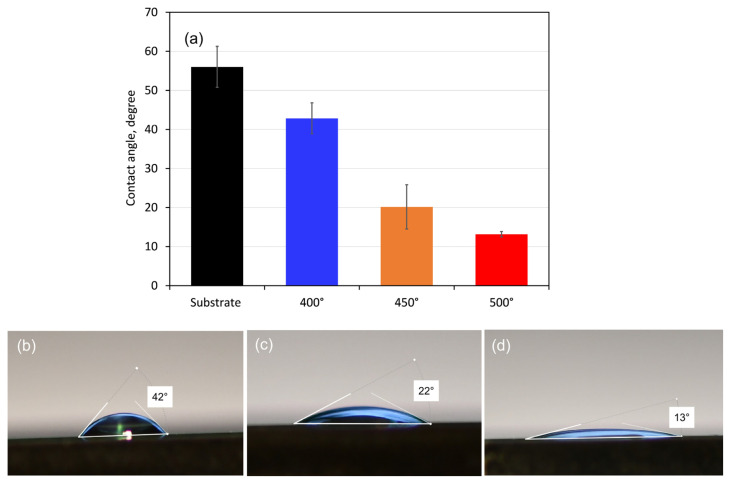
Mean contact angle values (**a**) with droplets of 5 µL SBF and side views of drops on the surface of (**b**) 400, (**c**) 450, and (**d**) 500 °C-coated samples. The bars indicate the standard deviation values.

Measurements of the coatings’ contact angles with SBF drops were used to assess their wettability. Since the ionic composition of the simulated body fluid (SBF) solution is comparable to that of human blood plasma, it was chosen as a test solution. Figure 5 displays the obtained contact angle values together with the distinctive form of SBF droplets on each type of coated surface. All sample surfaces had SBF droplet contact angles of less than 90°, indicating that the substrate and coating surfaces are hydrophilic. The contact angle values revealed that the coated surfaces are more hydrophilic (about 43, 20, and 13° for the 400, 450, and 500 °C-deposited films, respectively) than that of the pure alloy (about 56°). Therefore, the Ta oxide films will be more wetted in contact with SBF solution and are more likely to experience corrosion attack. However, when blood and biological fluids come into contact with hydrophilic surfaces, proteins adsorb in a conformation that reveals adhesion motifs and improves cell adherence, which aids in cell division and proliferation [49].

The measured contact angle values (Figure 5a) refer to the initial state of the compared tested material surface; however, with the progress of the electrochemical process and formation of corrosion layers, the wettability of the surfaces may change.

### 3.4. Electrochemical Tests

#### 3.4.1. Open Circuit Potentials

**Figure 6:**
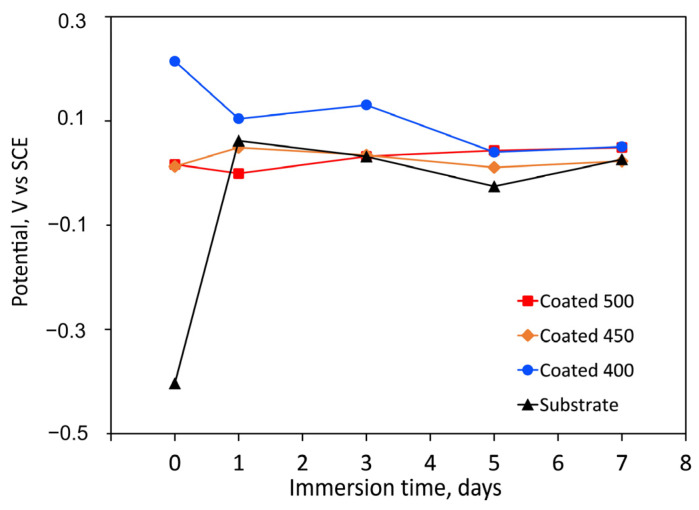
Change in time of the free corrosion potential (OCP vs. SCE) values, during the immersion period in SBF solution (at 37 °C) of the uncoated and coated samples.

The longevity of a prosthesis may be limited by surface degradation due to tissue responses to corrosive substances. Coatings’ resistance to corrosion can be influenced by several factors, including their thickness, porosity, wettability, composition, particle size, microstructure, etc. Figure 6 displays the change in OCP for both the bare substrate and the substrate coated with tantalum oxide films as a function of immersion time. Compared to the control, the amorphous coating displayed the highest positive OCP values. More positive OCP values are known to be linked to superior corrosion behaviour. Because of their less hydrophilic surfaces, the amorphous film had lower negative OCP values (reduced activity) than the more hydrophilic crystalline films (Figure 5). Due to their more negative OCP values, the crystalline coatings’ initial electrochemical activity ought to be higher. These findings might suggest that the a-Ta_2_O_5_ surface treatment improves the electrochemical resistance.

#### 3.4.2. Potentiodynamic Polarisation Tests

**Figure 7:**
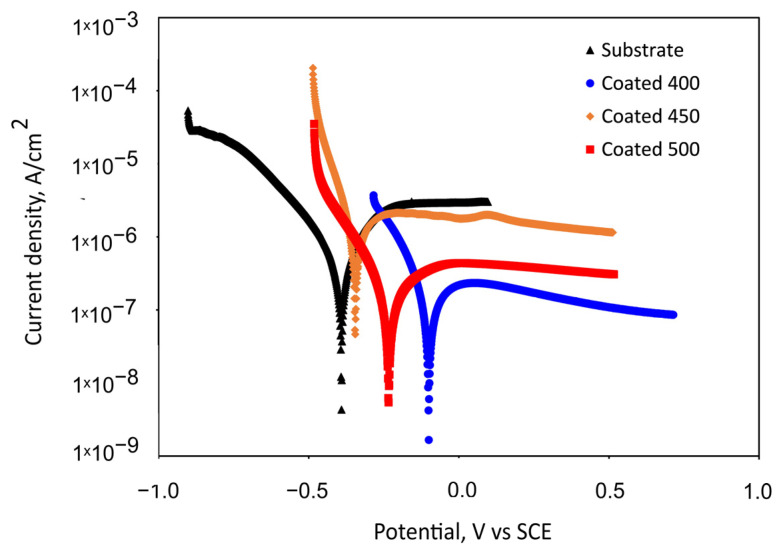
Representative potentiodynamic polarisation curves measured for the bare substrate and substrate with tantalum oxide coatings in SBF after 1 h of immersion.

The potentiodynamic polarisation curves for the Ti6Al4V substrate and the β-Ta_2_O_5_ coatings are shown in Figure 7. Table 3 summarises the values of the corrosion kinetic parameters, such as corrosion potentials (E_corr_) and corrosion current density (j_corr_), that were taken from the potentiodynamic curves using the Tafel extrapolation method, as well as the polarisation resistance (R_p_). The effectiveness of the coatings’ corrosion prevention was assessed using Equation (1) [50]:

(1) $$P.E.(\%)=\frac{I_{corr}^{0}-I_{corr}^{c}}{I_{corr}^{0}}\times 100$$

where $I_{corr}^{0}$ and $I_{corr}^{c}$, respectively, represent the corrosion current densities of the bare and coated states.

Figure 7 indicates the passive behaviour of all coated samples and bare alloy. For the substrate, an active–passive transition is seen following an initial range of cathodic de-passivation (Figure 7), with passivating currents of a few microamperes, which are commonly seen when passivating titanium and its alloys [51]. The polarisation curves reveal that the specimens coated at 450 °C and uncoated specimens’ onset of passivity occurs in the same potential region, suggesting that the formation of the passive oxide film appears to take place under comparable potential requirements and a similar mechanism. The small peak presence in the passive region of the 450 °C-deposited coating suggests a localised attack occurrence for this specimen in the SBF solution. The nanocrystalline film demonstrated lower R_p_ and higher j_corr_ values than the bare titanium alloy, which caused a lack of corrosion prevention effectiveness (Table 3). This electrochemical performance can be explained by the increased number of grain boundaries and other crystallographic imperfections, such as dislocations, vacancies, nanopores, stacking faults, etc.

Somewhat lower passivation current density values were observed for the 400 and 500 °C-coated samples. The a-Ta_2_O_5_ film has almost one order of magnitude higher R_p_ values than bare Ti6Al4V and crystalline films, indicating improved corrosion resistance and less metal dissolution in SBF during the first hour of immersion. It follows that the amorphous film is more resilient to corrosive attack. Table 3 shows that the 400 and 500 °C-coated samples indicate a shift in corrosion potential in a positive direction and a decrease in corrosion current density (j_corr_) in comparison with the bare substrate. Generally speaking, a material’s corrosion resistance is reduced when its corrosion current density is higher and its corrosion potential is lower. Consequently, these shifts suggest that these coated samples’ corrosion rates decreased. Although the density of the well-crystalline film may contribute to a decrease in the corrosion current or an increase in polarisation resistance (kinetic), it is unlikely to have an impact on the corrosion potential, as thermodynamics is more influenced by the stability or reactivity of the oxide film. The higher positive electrode potential of the amorphous film suggests a less reactive or more inert surface, which is also in line with the measurement of the contact angle, wettability, and phase composition. The a-Ta_2_O_5_ coating has improved corrosion resistance due to the lack of grain boundaries and, therefore, decreased electrochemical surface activity. Moreover, since the higher substrate temperature (500 °C) can increase the kinetic energy and mobility of atoms at the growing film surface, this extra energy would give the sputtered atoms a better chance of moving to the lowest energy state, thus decreasing the number of defects. The concentration of lattice distortions in the crystal structure diminishes as the size of the crystallite increases and the number of grain boundaries decreases at higher substrate temperatures. For that reason, the amorphous and well-crystalline Ta_2_O_5_ sputtered films have superior initial corrosion resistance over the 450 °C-deposited film and substrate, verifying that a chemical attack is less likely to affect their surfaces.

#### 3.4.3. Electrochemical Impedance Spectroscopy (EIS)

**Figure 8:**
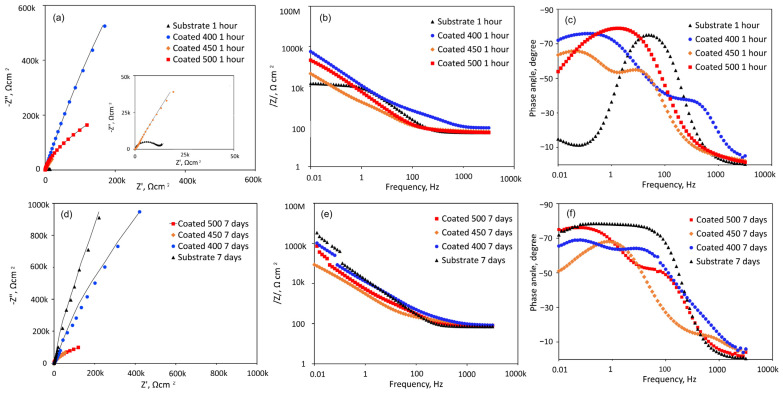
EIS analysis of the coated and uncoated samples immersed in SBF solution at 37 °C for 1 h and 7 days: (**a**,**d**) Nyquist diagrams; (**b**,**e**) Bode plots; (**c**,**f**) phase angle curves.

Figure 8 shows the EIS data obtained from the β-Ta_2_O_5_ coatings and Ti6Al4V substrate at their respective open-circuit potentials following one or 168 h of exposure, displayed as Nyquist, Bode impedance, and phase angle graphs. A magnified portion of the Nyquist plot in the lower range of values from Figure 8a is displayed in the inset.

Figure 8a shows that all Nyquist plots have a depressed capacitive semicircle shape. However, under the initial conditions, the semicircle for Ti6Al4V is more depressed than that of β-Ta_2_O_5_ coatings (Figure 8a inset). This suggests that the passive film formed on the coated surfaces is more protective than that of the bare Ti6Al4V in SBF. However, the diameters of the impedance arcs for the crystalline coatings decline with increasing exposure time (Figure 8d), but the capacitive semicircle diameters for the a-Ta_2_O_5_ coating remain mostly stable, suggesting a persistent deterioration in its resistance to corrosion. After 7 days of immersion, the impedance arc width for the bare alloy is consistently greater than that for all coated alloys, indicating that the β-Ta_2_O_5_ coatings corrode and dissolve faster than the bare sample, which exhibited capacitive behaviour typical of passive materials like Ti-based alloys.

The Bode magnitude plots display two distinct slopes (Figure 8b,e) over the frequency range under investigation. Within the first one, the impedance value |Z|, representing the electrolyte resistance (R_s_), is frequency-independent throughout the high-frequency range (1 × 10^3^ to 1 × 10^5^ Hz). The impedance value |Z|, which indicates the capacity reactance of the generated passive films, varies linearly with frequency in the low-frequency domain (1 × 10^−2^~1 × 10^3^ Hz). High corrosion resistance is indicated by high |Z| values at 0.01 Hz. Hence, initially, the samples with amorphous film display the highest overall resistance (Figure 8b), while after 7 days of immersion, this sample has been overtaken by the bare Ti6Al4V alloy (Figure 8e). The phase angle plots of all coated samples (Figure 8c,f) indicate the appearance of a two-time constant. For the coatings deposited at different temperatures, two maximum phase angles for each time constant appeared at various frequency ranges. Bode phase angle versus frequency plots of 400 and 500 °C-deposited coatings show a broad plateau with a maximum phase angle of roughly −75 and −80°, respectively, which is typical for an insulating oxide layer on a passive material’s surface [52]. The greater low-frequency zone explains the presence of a more compact and protective inner passive layer on the surface. This finding suggests that initially (Figure 8c) the coated samples’ surfaces are marginally more uniform than those of the uncoated specimens. Lower values are detected for the film deposited at 450 °C, probably due to higher reactivity and penetration of SBF solution into the film.

At middle-range frequencies, high phase angle values of roughly −70° are recorded for the initially immersed bare alloy (Figure 8c). The impact of the naturally occurring oxide coating on the alloy surface, which is made up of many oxide types with varying resistance to degradation under simulations, can be used to explain the nonuniform behaviour of the titanium alloy. Over the seven days of testing (Figure 8f), time constants of Ti6Al4V are associated with phase angle values of roughly −75° for medium- and low-frequency ranges due to the formation of passive barrier TiO_2_ layers when immersed in SBF.

All this suggests that the amorphous coating displays good corrosion resistance in SBF solution. With the increase in temperature up to 450 °C, the microstructure becomes inhomogeneous with nanocrystals and greater grain boundaries that are high-energy locations which increase corrosion rate [53]. The improved crystallinity, as confirmed by XRD and SEM analysis of the 500 °C-deposited film, displayed a more homogenous microstructure, which improved the stability of the passivation film to higher corrosion resistance than that of the nanocrystalline coating. However, its resistance deteriorated as the immersion time proceeded.

#### 3.4.4. Equivalent Electrical Circuit Models

The EIS spectra for corroding coated substrates can be further evaluated using numerical fitting in conjunction with the equivalent electrical circuit to quantify the corrosion behaviour.

**Figure 9:**
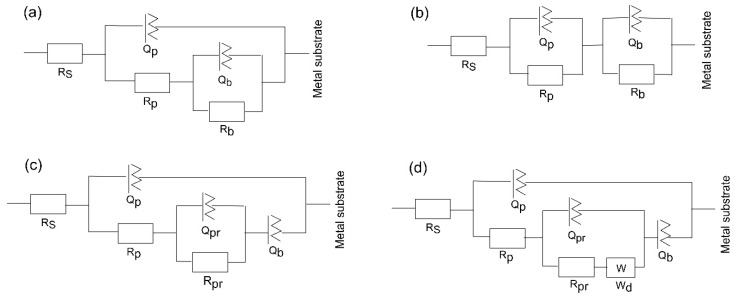
Equivalent circuit schemes used to match the impedance data of (**a**) substrate, (**b**) 400, (**c**) 450, and (**d**) 500 °C-deposited coatings for different times of immersion of the samples in SBF at 37 °C.

The substrate circuit was inferred to depict the electrochemical activity of a metal owning an unsealed porous layer covering it. When titanium was submerged in the medium for a shorter and longer period, the oxide layer changed into a Ti–TiO_2_ inner-TiO_2_ outer porous sealed layer [54]. In this scenario, in the equivalent circuit, as depicted in Figure 9a, there is the charge transfer resistance (R_p_) related to the electrolyte’s penetration via the coating’s pores or pinholes, and the inner barrier layer resistance (R_b_) together with the corresponding capacitance, Q_p_ and Q_b_, of the porous and intact (non-defective) layer. A frequency-dependent constant phase element (CPE or Q) with exponent “*n*” is used to replace an ideal capacitor to achieve a better match due to the electrode surface’s discontinuities and heterogeneities [55]. A deviation from the ideal capacitor/resistor is indicated by the exponent *n*, where *n* = 0 for a pure resistor and *n* = 1 for a pure capacitor.

The Bode phase angle graphs for amorphous coating (Figure 8c,f) are asymmetrical and have two maxima. This suggests that at least two time constants in the comparable circuit are interacting. To fit the EIS data for the amorphous film, an equivalent electrochemical circuit (EEC) model, represented as -R_s_(Q_p_R_p_)(Q_b_R_b_)- (Figure 9b) is introduced. The passive film is assumed to have a duplex layered structure with an inner compact layer and an outer porous layer with a sandwich structure in the model with two time constants. The outer passive layer in this EEC model is represented by the constant phase element (Q_p_) and resistance (R_p_), whereas the inner passive layer is associated with the constant phase element (Q_b_) and resistance (R_b_), in addition to the solution resistance (R_s_).

The EEC model of the substrate has been modified for the nanocrystalline and polycrystalline coatings deposited at 450 and 500 °C (Figure 9c,d) to describe sealed anodic oxide films. They account for the precipitation of some phases inside the porous film, which hinders the penetration of the electrolyte towards the metal substrate. The components Q_pr_ and R_pr_ in the circuit indicate the capacitance and resistance of precipitates inside the small micropores within the coatings. When one boundary imposes a fixed concentration for diffusing species, a cotangent diffusion element is commonly used to simulate finite-length diffusion [56] in micrometre-sized pores. Thus, a cotangent diffusion element (W) in series with the resistance of precipitates in the EEC of tested PVD crystalline coatings is fitted for the finite-length diffusion process within the pores.

Table 4 displays the electrical components fitted from the experimental EIS data. The sum of squares of residues, or Chi-square values (χ^2^), reaches a level near or exceeding 10^−3^~10^−4^. Therefore, each EEC model that was suggested for fitting the experimental data shows a good fit quality.

**Figure 10:**
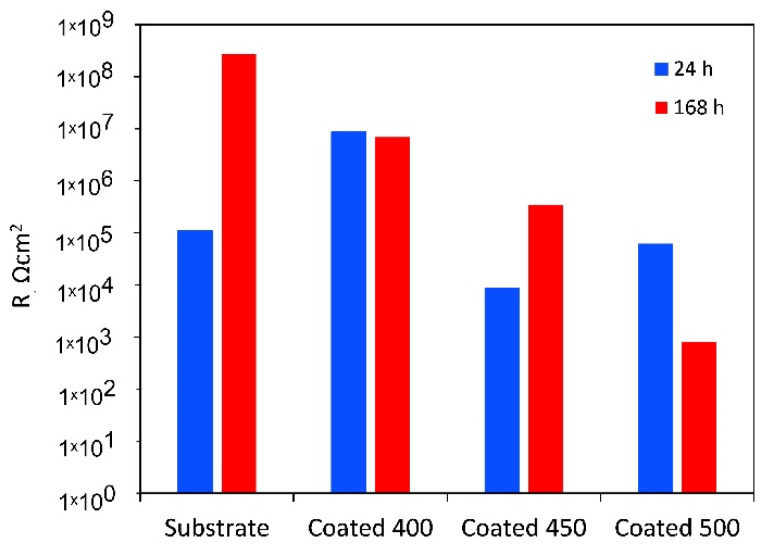
Polarisation resistance (R_p_ + R_b_) of the uncoated Ti6Al4V and β-Ta_2_O_5_ coatings.

For the amorphous coating, the R_b_ values are significantly greater than R_p_, as the fitting indicates that the inner barrier layer primarily regulates the tested samples’ corrosion resistance. It is believed that during the initial testing minutes, solution penetration into the inner layers of the coating will be negligible due to the higher contact angle with SBF. However, throughout the whole period that our experiments covered, slight changes were seen for the porous oxide layer capacitance (Q_p_), whereas the barrier layer capacitance (Q_b_) stayed almost unchanged (see Table 4). The greater diffusion through the outer layer may be the reason why the values of n_1_ are lower than those of n_2_. This finding suggests that as time increases, the coating acts as a barrier to prevent corrosion. The polarisation resistance values of a-Ta_2_O_5_ coating, calculated as the sum of the resistance of the porous passive film plus the resistance of the compact barrier layer (R_p_ + R_b_) [57] and presented in Figure 10, are near 10^7^ Ω cm^2^ and are almost unaffected by exposure duration.

The low R_p_ values after 1 h of immersion of the highly crystalline coating indicate that ions can travel more easily within its outer porous layer, perhaps due to increased porosities and higher surface wettability. Despite the higher deposition temperature, pinholes and voids, such as those detected in Figure 1c, are still present and could cause the coating’s decreased resistance to corrosion. These flaws contribute significantly to the start and spread of corrosion, which damages coatings in corrosive conditions [58]. After 7 days, the resistance of the porous layer increases while that of the small micropores in the compact part of the layer decreases, indicating that the precipitates cannot effectively block the diffusion. The aggressive Cl^−^ ions can easily penetrate these flaws, increasing the damage caused by electrochemical corrosion. In other words, metal dissolution proceeds through the coating because the salts deposited in the pores do not effectively impede it or are insufficient in quantity. As a result, the W value rises with time while the Q_pr_ value falls. The low coating thickness usually causes the rapid formation of localised galvanic cells and galvanic corrosion occurs. As a result, corrosion happens at two interfaces: the electrolyte substrate and the coating. The decline of R_p_ and R_b_ after 7 days of immersion is confirmed by the decrease in polarisation resistance (Figure 10), indicating that longer exposure times significantly worsen surface film disintegration.

The finer crystalline β-Ta_2_O_5_ coating can reduce the number of metal ions released from the coated system by somewhat impeding the electrochemical reactions occurring at the metal substrate contact, even though it is not an efficient anticorrosion layer. In general, corrosion resistance is higher for low Q and high n values than for high Q and low n values. The decreased Q_b_ and increased n_2_ values (Table 4) over 7 days of exposure point to reduced anodic site surface areas, which could cause corrosion. The temporal dependence of the fitted parameters R_pr_ and Q_pr_ (Table 4), which show the impacts of salt precipitation in the coating’s pores, supports this observation. After the immersion period, the polarisation resistance increases above 10^6^ Ω cm^2^ (Figure 10).

As mentioned earlier, when titanium alloy was submerged in a physiological solution, a double-layer titanium oxide film of a Ti–TiO_2_ inner–TiO_2_ outer porous unsealed layer formed [54]. Compared to R_b_, the resistance values for R_p_ are substantially greater. This outcome demonstrates that the outer layer of the passive film’s duplex-layer structure is more resilient than the inner layer. The examination of the R_p_ values with increasing exposure time presented in Table 4, which provides the electrolytic solution resistance inside the pores, also reveals the impact of precipitates partially obstructing the conductive ionic pathways in the pores after 7 days. The Q_p_, Q_b_, *n*_1_, and *n*_2_ values stayed almost unchanged, indicating that throughout the corrosion tests, the TiO_2_ film on the alloy surface consistently maintained its homogeneity and compactness. The polarisation resistance of Ti6Al4V rose from about 10^5^ to over 10^8^ Ω cm^2^ for the same immersion time (Figure 10).

### 3.5. Immersion Test in SBF Solution for 7 Days and Analysis of the Solution Chemistry

**Figure 11:**
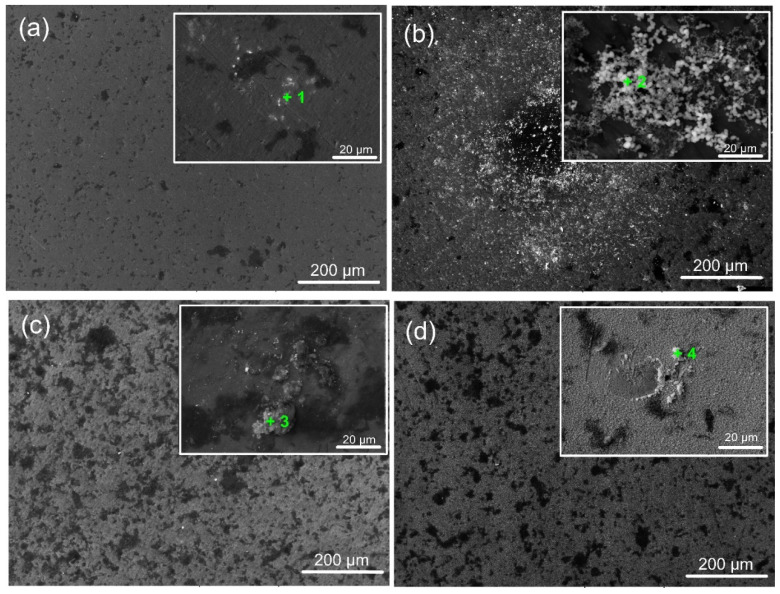
Representative SEM images of the bare and coated sample surfaces after 7 days of immersion in SFB solution (at 37 °C): (**a**) bare substrate; (**b**) 400 °C; (**c**) 450 °C; and (**d**) 500 °C-coated samples. The green marks indicate the EDS zones of interest.

Figure 11 displays micrographs of coated and untreated surfaces submerged in SBF for 7 days. The findings show inhomogeneous corrosion of Ti6Al4V at the microscale (Figure 11a), which may have been brought on by the development of micro-galvanic cells inside the metal’s multi-phase structure. Ta-coated surfaces exhibit surface deterioration like Ti6Al4V substrates with some corrosion pits. In contrast to the amorphous and well-crystalline Ta_2_O_5_ coating, the finer crystalline phases of the oxide film caused more severe corrosion (Figure 11c). This might be due to corrosive ions diffusing to the Ti/Ta_2_O_5_ contact via intrinsic flaws in the coating (such as voids and phase boundaries), which would cause under-film corrosion. The aggressive Cl^−^ ions can penetrate these flaws, increasing the damage caused by electrochemical corrosion. As a result, during exposure, craters steadily increase in size and quantity. The lack of grain boundaries for the amorphous film (Figure 11b) and superior integrity and fewer defects due to higher heating during deposition (Figure 11d) can afford fewer corrosion pits and craters to occur on a-Ta_2_O_5_ and well-crystallised β-Ta_2_O_5_ films, respectively. However, the corrosion test does not create an expansion force, causing the Ta-oxide covering to flake off. This is due to the good adhesion of all Ta oxide films on the titanium alloy surface, as confirmed by the scratch test results (Figure 4).

A biomaterial must be able to create an apatite layer in the human body that resembles bone to be considered bioactive. Calcium phosphate (CaP) mineralisation and precipitation on the specimens are indicators of their bioactivity, which is contingent upon the surface’s physicochemical characteristics [59]. After 7 days of incubation in SBF, the morphology of the coated specimens suggests different responses of the examined surfaces. The signal corresponding to the elements Ca and P, along with other desirable elements like Na and Mg, appeared in the relevant EDS spectra (Table 5), indicated with “+” in the corresponding images (Figure 11), confirming the presence of CaP on the surfaces following their immersion in SBF. The ion exchange between the fluid and the coated surface can explain the variance in the Ca/P ratio. Depending on their crystallinity, coatings soaked in simulated bodily fluid (SBF) frequently exhibit different bioactive behaviours [60].

**Figure 12:**
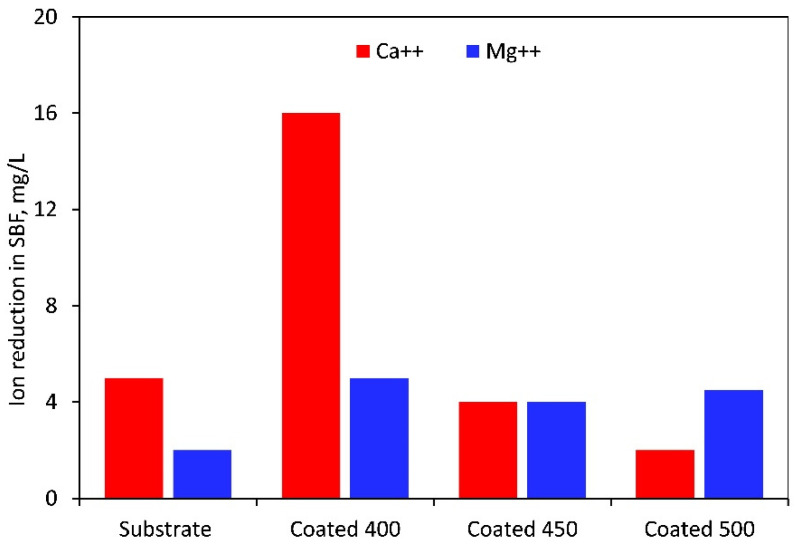
Magnesium and calcium ion reduction in SBF solution after 7 days of immersion of the uncoated and coated samples.

When soaked in SBF, the M-OH (M-metal) groups developed on the thin film surface start the CaP production process. The tantalum oxide layer in the SBF solution is gradually hydrated to create a hydrated oxide film containing Ta-OH groups. The SBF’s positively charged Ca^2+^, Mg^2+^, and Na^+^ ions are drawn to the surfaces of negatively charged metal oxide thin films, where they instantly mix with the negative phosphate ions to create a CaP layer and transform into stable apatite. Apatite production resulted from the enormous spontaneous consumption of Ca^2+^ ions and PO_4_^3−^ ions from the SBF during the first nucleation of CaP [61,62]. Figure 12 displays the Mg^2+^ and Ca^2+^ concentrations that were determined by photometry due to the precipitation of magnesium during the immersion test. The value recorded at t = 0 min, or before the samples were submerged, was taken into account for recalculating the reduction in Mg^2+^ and Ca^2+^ concentration. As indicated in Figure 12, in contrast to the rest of the samples, the highest amounts of Ca^2+^ and Mg^2+^ ions were consumed from SBF after immersion of the 400 °C-coated sample. Therefore, more Ta hydroxide groups combine with calcium and phosphate ions to form a layer of calcium phosphate on this film surface. The higher amount of oxygen in the coating may cause the observed phenomenon. The physical evidence of the precipitation and formation of a bone-like apatite is the granular-like texture observed on the surface of the coating deposited at 400 °C (Figure 11b). This layer may prevent the substrate from degrading and produce a capacitive response [63]. Similar precipitates were observed on the surface of the other samples, but with different morphology, less dense, and limited in quantity. These facts completely coincide with and confirm the EIS results. However, based on the Ca/P ratio value (Table 5) acquired in 7 days of immersion, Ca-deficient layers (less than the stoichiometric (1.67) hydroxyapatite) are formed on all sample surfaces.

The change in pH values of the SBF solution after the first 24 h (1 day), 3 days, and 7 days of immersion of the samples is compared in Table 6. There was a slight decrease in pH during the first day (24 h) of immersion due to acidic hydroxides (M-OH → M-O^−^ + H^+^) and protonated base (M-OH_2_^+^ → M-OH^−^ + H^+^) deprotonation [64]. Jemat et al. reported similar findings [65]. After that, the pH values of the SBF were more alkaline for the coated samples compared to those of the uncoated surface. This tendency of slow increase, along with the immersion period, was maintained until the end of the test (7 days of immersion) for all samples. The M-OH^−^ groups’ negative charges are the chemical stimulus needed to allow apatite to form since they draw calcium ions out of the solution to create calcium titanate, which serves as an apatite deposit nucleation point [66]. As a result, the surface availability of these Ti-OH^−^ groups dictates how well the surface forms apatite. Compared to the other coatings and bare substrate, the slightly higher pH values of the 400 °C-deposited coatings in the solution indicated superior apatite-forming capabilities. Therefore, the 400 °C-deposited amorphous coating consists of a bilayer structure, containing an exterior layer of deposited calcium phosphate that can promote osseointegration [67] and an inner barrier layer of Ta_2_O_5_ that guards against corrosion. Since CaP precipitation causes HAp to develop, its presence can initiate bone formation with a higher healing rate [68].

### 3.6. Shortcomings of the Research

The study’s findings show that depositing β-Ta_2_O_5_ coatings could be a future surface treatment for titanium implants that enhances the mechanical and long-term stability, but with some additional improvements. The titanium alloy’s durability and corrosion resistance can be further enhanced by depositing a titanium oxide film at higher than 500 °C substrate temperature, thickening the β-Ta_2_O_5_ film, or obtaining multilayer Ta_2_O_5_/Ta or Ta_2_O_5_/TaN coatings. Additionally, it is known that in both reduced and oxidised forms, tantalum exhibits minimal unfavourable biological response and is extremely resistant to chemical attack [69]. Nonetheless, to assess how such formed surfaces interact with live tissues, in vitro and in vivo biological examinations are necessary.

## 4. Conclusions

β-Ta_2_O_5_ coatings were successfully deposited onto Ti6Al4V alloy substrates using magnetron sputtering at 400, 450, and 500 °C substrate temperatures. Our results show that increasing the deposition temperature transforms the initially amorphous films into well-crystallised β-Ta_2_O_5_ structures without significantly changing the film thickness. Higher temperatures also improved the adhesion of the oxide coatings to the substrate and their hardness values. The application of tantalum oxide coatings reduced the coefficient of friction of the Ti6Al4V alloy.

Electrochemical tests indicated that both amorphous and crystalline films improved corrosion resistance, as evidenced by the reduced corrosion current densities observed in the coatings deposited at 400 and 500 °C compared to the uncoated alloy. Electrochemical impedance spectroscopy (EIS) further supported these findings: the initial polarisation resistance of the amorphous Ta_2_O_5_ coatings reached approximately 10^7^ Ω·cm^2^—two orders of magnitude higher than bare Ti6Al4V. However, over time, the corrosion resistance of the crystalline coatings declined due to the presence of micro-defects. EIS analysis revealed that the amorphous film acted as an effective barrier against the penetration of simulated body fluid (SBF), while the well-crystallised film deteriorated more rapidly under the same conditions.

Bioactive evaluations showed that the amorphous coating performed very well in SBF, facilitating the formation of a calcium-deficient apatite layer after just seven days of immersion. Considering its mechanical performance, strong adhesion to the Ti alloy interface, and favourable in vitro apatite-formation properties, β-Ta_2_O_5_ coatings hold significant promise as a next-generation alternative to conventional coatings for titanium-based orthopaedic implants.

Our next objective is to further optimise the coatings’ architecture and deposition conditions so that the tantalum oxide film meets all requirements for long-term bone healing devices.

## Figures and Tables

**Table 1:** Relative tantalum and oxygen content (at% ± standard deviation (SD)) in the examined coatings.

Sample	Ta, at% ± SD	O, at% ± SD	O/Ta
Coated at 400 °C	14.5 ± 2.2	61.3 ± 4.5	4.2
Coated at 450 °C	15.4 ± 1.9	57.5 ± 3.6	3.7
Coated at 500 °C	17.3 ± 0.7	46.9 ± 3.2	2.7

**Table 2:** Microhardness, critical adhesion forces, and coefficient of friction values (±standard deviation) of the substrate and coated samples at different temperatures.

Sample	HK_0.005_ (kgf mm^−2^)	F_c_ (N)	COF
Substrate	561.5 ± 14	-	0.39 ± 0.02
Coated at 400 °C	1038 ± 20	8.6 ± 0.3	0.34 ± 0.04
Coated at 450 °C	1197.5 ± 63	9.9 ± 0.2	0.31 ± 0.06
Coated at 500 °C	1659.3 ± 67	20 ± 1.0	0.22 ± 0.03

**Table 3:** Corrosion potential (E_corr_), corrosion current density (j_corr_), polarisation resistance (R_p_), and protection efficacy (P.E.) values obtained for the substrate and coated samples.

Sample	E_corr_ (mV vs. SCE)	j_corr_ (10^−6^ A cm^−2^)	R_p_ (kΩ)	P.E. (%)
Ti6Al4V	−391	1.4	20.5	-
Coated at 400 °C	−100	0.2	178	85.7
Coated at 450 °C	−345	2.1	15.4	-
Coated at 500 °C	−234	0.5	37.3	64.3

**Table 4:** Electrochemical parameters derived via numerical fitting for uncoated Ti6Al4V and β-Ta_2_O_5_ coatings deposited at different temperatures.

	Sample	Ti6Al4V		Ta_2_O_5_ 400 °C		Ta_2_O_5_ 450 °C		Ta_2_O_5_ 500 °C	
Time		24 h	168 h	24 h	168 h	24 h	168 h	24 h	168 h
Q_p_, (Ω^−1^cm^−2^s^n^)		1.3 × 10^−5^	1.1 × 10^−5^	2.2 × 10^−5^	2.3 × 10^−4^	1.5 × 10^−4^	4.6 × 10^−5^	1.4 × 10^−5^	1.0 × 10^−5^
n_1_		0.91	0.91	0.64	0.45	0.69	0.60	0.88	0.85
R_p_, (Ωcm^2^)		1.1 × 10^5^	2.7 × 10^8^	7.2 × 10^2^	1.9 × 10^4^	8.8 × 10^3^	9.2 × 10^1^	4.4 × 10^1^	1.2 × 10^2^
Q_b_, (Ω^−1^cm^−2^s^n^)		8.7 × 10^−4^	1.1 × 10^−4^	1.9 × 10^−5^	1.8 × 10^−5^	1.0 × 10^−2^	3.7 × 10^−5^	3.8 × 10^−5^	5.2 × 10^−6^
n_2_		0.53	0.52	0.85	0.84	0.76	1	0.86	0.86
R_b_/R_pr_, (Ωcm^2^)		5.1 × 10^−9^	9.2 × 10^−6^	8.7 × 10^6^	6.9 × 10^6^	4.9 × 10^−1^	3.5 × 10^5^	6.1 × 10^4^	6.9 × 10^2^
Q_pr_, (Ω^−1^cm^−2^s^n^)		-	-	-	-	4.1 × 10^−5^	4.3 × 10^−4^	2.1 × 10^−3^	3.4 × 10^−5^
n_3_		-	-	-	-	1	0.47	1	1
Q_pr_, (Ω^−1^cm^−2^s^0.5^)		-	-	-	-	-	-	2.1 × 10^−3^	1.3 × 10^−4^
W (s^0.5^)		-	-	-	-	-	-	5.7 × 10^−2^	1.65

**Table 5:** Element content (at%) of the points highlighted in the insets of Figure 11.

Sample	Ca	Mg	Na	P	O	Ca/P Ratio
Ti6Al4V	5.4	0.15	1.47	3.6	92.1	1.50
Coated at 400 °C	6.7	0.4	1.5	4.4	79.2	1.52
Coated at 450 °C	6.3	0.3	0.96	4.56	81.3	1.38
Coated at 500 °C	1.3	0.3	1.4	0.9	82.5	1.44

**Table 6:** Changes in pH of SBF (at 37 °C) after 1 day and 7 days of immersion of the uncoated and coated samples.

Samples	1 Day	3 Days	7 Days
Substrate	7.43	7.53	7.54
Coated at 400 °C	7.07	7.58	7.59
Coated at 450 °C	7.20	7.53	7.54
Coated at 500 °C	7.11	7.56	7.57

## Data Availability

The original contributions presented in the study are included in the article; further inquiries can be directed to the corresponding author.

## References

[1] Prasad S., Ehrensberger M., Gibson M.P., Kim H., Monaco E.A. (2015). Biomaterial properties of titanium in dentistry. J. Oral Biosci..

[2] Geetha M., Singh A.K., Asokamani R., Gogia A.K. (2009). Ti based biomaterials, the ultimate choice for orthopaedic implants—A review. Prog. Mater. Sci..

[3] Kuromoto N.K., Simão R.A., Soares G.A. (2007). Titanium oxide films produced on commercially pure titanium by anodic oxidation with different voltages. Mater. Charact..

[4] Portan D.V., Kroustalli A.A., Deligianni D.D., Papanicolaou G.C. (2012). On the biocompatibility between TiO_2_ nanotubes layer and human osteoblasts. J. Biomed. Mater. Res. A.

[5] Javed F., Romanos G.E. (2009). Impact of diabetes mellitus and glycemic control on the osseointegration of dental implants: A systematic literature review. J. Periodontol..

[6] Nine M.J., Choudhury D., Hee A.C., Mootanah R., Osman N.A.A. (2014). Wear debris characterization and corresponding biological response: Artificial hip and knee joints. Materials.

[7] Rae T. (1986). The biological response to titanium and titanium-aluminium-vanadium alloy particles. Biomaterials.

[8] Khadija G., Saleem A., Akhtar Z., Naqvi Z., Gull M., Masood M., Mukhtar S., Batool M., Saleem N., Rasheed T. (2018). Short term exposure to titanium, aluminum and vanadium (Ti 6Al 4V) alloy powder drastically affects behavior and antioxidant metabolites in vital organs of male albino mice. Toxicol. Rep..

[9] Oliveira V.M.C.A., Aguiar C., Vazquez A.M., Robin A., Barboza M.J.R. (2014). Improving corrosion resistance of Ti–6Al–4V alloy through plasma-assisted PVD deposited nitride coatings. Corros. Sci..

[10] Meng F., Li Z., Liu X. (2013). Synthesis of tantalum thin films on titanium by plasma immersion ion implantation and deposition. Surf. Coat. Technol..

[11] Niinomi M. (2008). Mechanical biocompatibilities of titanium alloys for biomedical applications. J. Mech. Behav. Biomed. Mater..

[12] Soro N., Attar H., Brodie E., Veidt M., Molotnikov A., Dargusch M.S. (2019). Evaluation of the mechanical compatibility of additively manufactured porous Ti–25Ta alloy for load-bearing implant applications. J. Mech. Behav. Biomed. Mater..

[13] Weisman S. (1968). Metals for implantation in the human body. Ann. N. Y. Acad. Sci..

[14] Shiri S., Zhang C., Odeshi A., Yang Q. (2018). Growth and characterization of tantalum multilayer thin films on CoCrMo alloy for orthopaedic implant applications. Thin Solid Films.

[15] Rahmati B., Sarhan A.A.D., Zalnezhad E., Kamiab Z., Dabbagh A., Choudhury D., Abas W.A.B.W. (2016). Development of tantalum oxide (Ta-O) thin film coating on biomedical Ti-6Al-4V alloy to enhance mechanical properties and biocompatibility. Ceram. Int..

[16] Rahmati B., Sarhan A.A.D., Basirun W.J., Abas W.A.B.W. (2016). Ceramic tantalum oxide thin film coating to enhance the corrosion and wear characteristics of Ti6Al4V alloy. J. Alloys Compd..

[17] Almeida Alves C.F., Cavaleiro A., Carvalho S. (2016). Bioactivity response of Ta1-xOx coatings deposited by reactive DC magnetron sputtering. Mater. Sci. Eng. C.

[18] Xu G., Shen X., Hu Y., Ma P., Cai K. (2015). Fabrication of tantalum oxide layers onto titanium substrates for improved corrosion resistance and cytocompatibility. Surf. Coat. Technol..

[19] Metikoš-Hukovi’c M., Kwokal A., Piljac J. (2003). The influence of niobium and vanadium on passivity of titanium-based implants in physiological solution. Biomaterials.

[20] Beline T., da Silva J.H.D., Matos A.O., Azevedo Neto N.F., de Almeida A.B., Nociti Júnior F.H., Leite D.M.G., Rangel E.C., Barão V.A.R. (2019). Tailoring the synthesis of tantalum-based thin films for biomedical application: Characterization and biological response. Mater. Sci. Eng. C..

[21] Zhang X.-M., Li Y., Gu Y.-X., Zhang C.-N., Lai H.-C., Shi J.-Y. (2019). Ta-Coated Titanium Surface with Superior Bacteriostasis And Osseointegration. Int. J. Nanomed..

[22] Chang Y.Y., Huang H.L., Chen H.J., Lai C.H., Wen C.Y. (2014). Antibacterial properties and cytocompatibility of tantalum oxide coatings. Surf. Coat. Technol..

[23] Sun Y.S., Chang J.H., Huang H.H. (2013). Corrosion resistance and biocompatibility of titanium surface coated with amorphous tantalum pentoxide. Thin Solid Films.

[24] Zein El Abedin S., Welz-Biermann U., Endres F. (2005). A study on the electrodeposition of tantalum on NiTi alloy in an ionic liquid and corrosion behaviour of the coated alloy. Electrochem. Commun..

[25] Balla V.K., Banerjee S., Bose S., Bandyopadhyay A. (2010). Direct laser processing of a tantalum coating on titanium for bone replacement structures. Acta Biomater..

[26] Anbalagan A.K., Cummings R., Zhou C., Mun J., Stanic V., Jordan-Sweet J., Yao J., Kisslinger K., Weiland C., Nykypanchuk D. (2025). Revealing the Origin and Nature of the Buried Metal-Substrate Interface Layer in Ta/Sapphire Superconducting Films. Adv. Sci..

[27] Bend A., Kandadai V.A.S., Petersen J.B., Jasthi B.K. (2025). Effect of deposition pressure on the microstructure, mechanical, and corrosion properties of tantalum nitride thin films deposited by reactive pulsed laser deposition. Vacuum.

[28] Tian X., Ding Y., Chai G., Tang Y., Lei R., Jia G., Zhang Y., Li J., Zhou Y., Wang X. (2024). Plasma-Enhanced Atomic Layer Deposition of Amorphous Tantalum Thin Films for Copper Interconnects Using an Organometallic Precursor. Adv. Mater. Technol..

[29] Yang X., Gao M., Liu Y., Li J., Huang Y., Wang G. (2022). Superior corrosion resistance of high-temperature Ir–Ni–Ta–(B) amorphous alloy in sulfuric acid solution. Corros. Sci..

[30] Song B., Hua Y., Zhou C., Li Y., Yang L. (2022). Fabrication and anticorrosion behavior of a bi-phase TaNbHfZr/CoCrNi multilayer coating through magnetron sputtering. Corros. Sci..

[31] Khalil-Allafi J., Daneshvar H., Safavi M.S., Khalili V. (2021). A survey on crystallization kinetic behavior of direct current magnetron sputter deposited NiTi thin films. Phys. B Condens. Matter.

[32] Bright T.J., Watjen J.I., Zhang Z.M., Muratore C., Voevodin A.A., Koukis D.I., Tanner D.B., Arenas D.J. (2013). Infrared optical properties of amorphous and nanocrystalline Ta_2_O_5_ thin films. J. Appl. Phys..

[33] Li J., Liu W., Wei Y., Yan Y. (2022). Effect of Oxygen Content on the Properties of Sputtered TaOx Electrolyte Film in All-Solid-State Electrochromic Devices. Coatings.

[34] Wu S.J., Houng B., Huang B. (2009). Effect of growth and annealing temperatures on crystallization of tantalum pentoxide thin film prepared by RF magnetron sputtering method. J. Alloys Compd..

[35] Narayanan R., Seshadri S.K., Kwon T.Y., Kim K.H. (2008). Calcium phosphate-based coatings on titanium and its alloys. J. Biomed. Mater. Res. B.

[36] Macionczyk F., Gerold B., Thull R. (2001). Repassivating tantalum/tantalum oxide surface modification on stainless steel implants. Surf. Coat. Technol..

[37] Sahoo K.K., Pradhan D., Ghosh S.P., Gartia A., Kar J.P. (2024). Modulation of electrical properties of sputtered Ta_2_O_5_ films by variation of RF power and substrate temperature. Phys. Scr..

[38] Yamada A., Sang B., Konagai M. (1997). Atomic layer deposition of ZnO transparent conducting oxides. Appl. Surf. Sci..

[39] Almeida Alves C.F., Mansilla C., Pereira L., Paumier F., Girardeau T., Carvalho S. (2018). Influence of magnetron sputtering conditions on the chemical bonding, structural, morphological and optical behavior of Ta_1−x_O_x_ coatings. Surf. Coat. Technol..

[40] Ohring M. (2002). Materials Science of Thin Films: Deposition and Structure.

[41] Martin N., Cote J.-M., Gavoille J., Potin V. (2023). Tantalum Oxide Thin Films Sputter-Deposited by Oxygen Gas Pulsing. Coatings.

[42] Favaro G., Milotti V., Diaz Riega D.A., Busdon N., Bazzan M., Granata M., Hofman D., Michel C., Pinard L., Conti L. (2024). Reduction of mechanical losses in ion-beam sputtered tantalum oxide thin films via partial crystallization. Class. Quantum Grav..

[43] Heinrichs J., Jarmar T., Wiklund U., Engqvist H. (2008). Physical Vapour Deposition and Bioactivity of Crystalline Titanium Dioxide Thin Films. Engqvist Artif. Organs Trends Biomater. Artif. Organs..

[44] Guisbiers G., Herth E., Buchaillot L., Pardoen T. (2010). Fracture toughness, hardness, and Young’s modulus of tantalum nanocrystalline films. Appl. Phys. Lett..

[45] Xu J., Hua W., Xie Z.-H., Munroe P. (2016). Reactive-sputter-deposited β-Ta_2_O_5_ and TaON nanoceramic coatings on Ti–6Al–4V alloy against wear and corrosion damage. Surf. Coat. Technol..

[46] Li H., Ding Y., Hu X., Li W., Ding Z. (2024). A comparative study of TiO_2_, Ta_2_O_5_ and Nb_2_O_5_ coated Ti6Al4V titanium alloy for biomedical applications. Ceram. Int..

[47] Davis C.A. (1993). A simple model for the formation of compressive stress in thin films by ion bombardment. Thin Solid Films.

[48] Wang L., Aversa R., Houa Z., Tian J., Liang S., Ge S., Chen Y., Perrotta V., Apicella A., Apicella D. (2021). Bioresorption control and biological response of magnesium alloy AZ31 coated with poly-β-hydroxybutyrate. Appl. Sci..

[49] Gittens R.A., Scheideler L., Rupp F., Hyzy S.L., Geis-Gerstorfer J., Schwartz Z., Boyan B.D. (2014). A review on the wettability of dental implant surfaces II: Biological and clinical aspects. Acta Biomater..

[50] Hirpara J., Chawla V., Chandra R. (2020). Investigation of tantalum oxynitride for hard and anti-corrosive coating application in diluted hydrochloric acid solutions. Mater. Today Commun..

[51] Burstein G.T., Souto R.M. (1995). Observations of localised instability of passive titanium in chloride solution. Electrochim. Acta.

[52] Bose S., Pathak L.C., Singh R. (2018). Response of boride coating on the Ti-6Al-4V alloy to corrosion and fretting corrosion behavior in Ringer’s solution for bio-implant application. Appl. Surf. Sci..

[53] Prithivirajan S., Nyahale M.B., Naik G.M., Narendranath S., Prabhu A., Rekha P.D. (2021). Bio-corrosion impacts on mechanical integrity of ZM21 Mg for orthopaedic implant application processed by equal channel angular pressing. J. Mater. Sci. Mater. Med..

[54] Pan J., Leygraf C., Thierry D., Ektessabi A.M. (1997). Corrosion resistance for biomaterial applications of TiO_2_ films deposited on titanium and stainless steel by ion-beam-assisted sputtering. J. Biomed. Mater. Res..

[55] Ryl J., Wysocka J., Slepski P., Darowicki K. (2016). Instantaneous impedance monitoring of synergistic effect between cavitation erosion and corrosion processes. Electrochim. Acta.

[56] Boukamp B.A. (1986). Nonlinear Least Squares Fit Procedure for Analysis of Immittance Data of Electrochemical Systems. Solid State Ionics.

[57] Moreto J.A., Marino C.E.B., Bose Filho W.W., Rocha L.A., Fernandes J.C.S. (2014). SVET, SKP and EIS study of the corrosion behaviour of high strength Al and Al-Li alloys used in aircraft fabrication. Corros. Sci..

[58] Ahmed M.S., Munroe P., Jiang Z.-T., Zhao X., Rickard W., Zhou Z., Li L.K.Y., Xie Z. (2011). Corrosion behaviour of nanocomposite TiSiN coatings on steel substrates. Corros. Sci..

[59] Zhang J., Dai C., Wei J., Wen Z., Zhang S., Chen C. (2013). Degradable behavior and bioactivity of micro-arc oxidized AZ91D Mg alloy with calcium phosphate/chitosan composite coating in m-SBF. Colloids Surf. B.

[60] Stiegler N., Bellucci D., Bolelli G., Cannillo V., Gadow R., Killinger A., Lusvarghi L., Sola A. (2012). Explore all metrics High-velocity suspension flame sprayed (HVSFS)hydroxyapatite coatings for biomedical applications. J Therm. Spray Technol..

[61] Rajan S.T., Das M., Arockiarajan A. (2022). In vitro biocompatibility and degradation assessment of tantalum oxide coated Mg alloy as biodegradable implants. J. Alloys Compd..

[62] Cai C., Wang X., Li B., Dong K., Shen Y., Li Z., Shen L. (2021). Fabrication of hydroxyapatite/tantalum composites by pressureless sintering in different atmosphere. ACS Omega.

[63] Hee A.C., Zhao Y., Jamali S.S., Bendavid A., Martin P.J., Guo H. (2019). Characterization of tantalum and tantalum nitride films on Ti6Al4V substrate prepared by filtered cathodic vacuum arc deposition for biomedical applications. Surf. Coat. Technol..

[64] Karimi S., Nickchi T., Alfantazi A. (2011). Effects of bovine serum albumin on the corrosion behaviour of AISI 316L, Co–28Cr–6Mo, and Ti–6Al–4V alloys in phosphate buffered saline solutions. Corros. Sci..

[65] Jemat A., Ghazali M.J., Razali M., Otsuka Y., Rajabi A. (2018). Effects of TiO_2_ on microstructural, mechanical properties and in-vitro bioactivity of plasma sprayed yttria stabilised zirconia coatings for dental application. Ceram Intern..

[66] Pereira B.L., Tummler P., Marino C.E.B., Soares P.C., Kuromoto N.K. (2014). Titanium bioactivity surfaces obtained by chemical/electrochemical treatments. Matéria.

[67] Xu J., Fu T., Lyu Y., Munroe P., Xie Z. (2018). In vitro biocompatibility of a nanocrystalline β-Ta_2_O_5_ coating for orthopaedic implants. Ceram. Int..

[68] Amaravathy P., Sowndarya S., Sathyanarayanan S., Rajendran N. (2014). Novel sol gel coating of Nb_2_O_5_ on magnesium alloy for biomedical applications. Surf. Coat. Technol..

[69] Black J. (1994). Biological Performance of Tantalum. Clin. Mater..

